# Amyloid-Beta 1-42 Cross-Reactive Antibody Prevalent in Human Sera May Contribute to Intraneuronal Deposition of A-Beta-P-42

**DOI:** 10.1155/2018/1672568

**Published:** 2018-06-21

**Authors:** Aristo Vojdani, Elroy Vojdani

**Affiliations:** ^1^Immunosciences Lab., Inc., 822 S. Robertson Blvd., Ste. 312, Los Angeles, CA 90035, USA; ^2^Department of Preventive Medicine, Loma Linda University School of Medicine, 24785 Stewart St., Evans Hall, Ste. 111, Loma Linda, CA 92354, USA; ^3^Regenera Medical, 11860 Wilshire Blvd., Ste. 301, Los Angeles, CA 90025, USA

## Abstract

Antibodies against many neural antigens are detected in the sera of both patients with Alzheimer's disease (AD) and some healthy individuals. Blood-brain barrier dysfunction could make it possible for brain-reactive autoantibodies to reach the brain, where they can react with amyloid ß peptide (AßP). The origin of these autoreactive antibodies in the blood is unclear. The goals of this study were as follows: (1) to examine the immune reactivity of anti-AßP-42 with 22 neuronal and other associated antigens, some of which are involved in the pathophysiology of AD; (2) to classify antibodies to these 22 different antigens into those that cross-react with AßP-42 and those that do not; (3) to determine whether these antibodies react with BBB proteins, nerve growth factors, and enteric neuronal antigens. Using monoclonal AßP-42 antibody and ELISA methodology, we found that the antibody was highly reactive with Aß protein, tau protein, presenilin, rabaptin-5, *β*-NGF, BDNF, mTG, and enteric nerve. The same antibody produced equivocal to moderate reactions with glutamate-R, S100B, AQP4, GFAP, MBP, *α*-synuclein, tTG-2, and tTG-3, and not with the rest. These antibodies were also measured in blood samples from 47 AD patients and 47 controls. IgG antibodies were found to be elevated against AßP-42 and many other antigens in a significant percentage of controls. Overall, the mean OD values were significantly higher against 9/23 tested antigens (*p* <0.001) in the samples with AD. We were indeed able to classify the detected neuronal antibodies into those that cross-react with AßP-42 and those that do not. Our main finding is that although these antibodies may be harmless in a subgroup of controls, in individuals with compromised BBBs these antibodies that cross-react with AßP-42 can reach the brain, where their cross-reactivity with AßP-42 may contribute to the onset and progression of AD, and perhaps other neurodegenerative disorders.

## 1. Background

It is commonly accepted that amyloid-*β* (A*β*) is a key protein in Alzheimer's disease (AD). The buildup of these proteins in the brain is considered a defining feature of AD as they are found in 60% of Alzheimer's cases [1]. The exact role of the protein and its antibody, however, is a matter of some dispute, as different studies have shown both detrimental and protective properties for them [2]. Bourgade showed that A*β*P 1-40 and A*β*P 1-42 acted directly to prevent the entry of HSV-1 into cells [3, 4], while Kumar found that A*β*P demonstrated antimicrobial actions as part of the innate immune system [5].

Oddly enough, anti-AßP-42 antibodies can also be found in the sera of healthy human individuals. In fact, other brain-reactive autoantibodies have also been found to be nearly ubiquitous in human sera [6, 7]. The question, then, is, when do these autoantibodies become pathogenic to their host, and how? To answer this question we must examine the nature of these known reactive autoantibodies and study how they interact with and affect each other. In particular we must focus on their relationship with AßP-42, a key element and feature in Alzheimer's disease.

To this end we set out to study the immune reactivity of anti-AßP-42 with 22 neuronal and other associated antigens, some of which are involved in the pathophysiology of AD. Antibodies against a variety of neural antigens such as amyloid ß proteins and peptides (1-42), tau protein, asialoganglioside GM_1_, S100B, glial fibrillary acidic protein (GFAP), rabaptin-5 (rab-5), adenosine triphosphate synthase (ATP-synthase), myelin basic protein (MBP), and many others known as autoantigens in Alzheimer's disease (AD) are detected in the sera or cerebrospinal fluids (CSF) of patients with AD [2, 8]. These antibodies are also found at much lower levels in the blood of many healthy individuals [7]. Although these ubiquitous autoantibodies can be classified or categorized in many different ways, for our purposes we divided them into four general groups.

AßP-42, tau protein, *α*-synuclein, asialoganglioside GM_1_, GFAP, rab-5, ATP-synthase, MBP, and their antibodies have been linked with neurodegeneration and diseases such as AD, Parkinson's disease (PD), and multiple sclerosis (MS) [2, 7–14]. Aquaporin-4 (AQP4) and S100B have been linked to increased permeability of the blood-brain barrier (BBB), neuromyelitis optica, and dementia, among others [2, 13, 14]. Glutamate receptor (glutamate-R), dopamine receptors 1,2,* N*-methyl-D-aspartate receptor (NMDAR), and glutamic acid decarboxylase 65 (GAD-65) are associated with neuroautoimmunity, including Sydenham's chorea and gluten ataxia [2, 15–18]. Transglutaminases (tTGs) are a group of enzymes that catalyze various posttranslational modifications of glutamine residues in proteins and peptides [19]. Tissue transglutaminases such as tTG-2 and tTG-3 are known as endogenous transglutaminases. Antibodies against tTG-2, tTG-3, and tTG-6 are detected in patients with celiac disease (CD), dermatitis herpetiformis, and gluten ataxia [18, 20–22]. The exogenous microbial transglutaminase (mTG) is a universal protein cross-linker and translational modifier of peptides made from* Streptoverticillium mobaraense *that imitates the function of endogenous tTGs [23]. It is used industrially as meat glue to bind lesser cuts of meat and other kinds of food together [24]. Studies indicate that the widespread use of mTG in different industries has contributed to the surge of CD and nonceliac gluten sensitivity (NCGS) [23, 24].

Elevation in the levels of antibodies against these distinct molecular antigens suggests that autoimmune components could play a role in AD [8, 12]. This elevation is detectable in blood, which means that these antibodies could be developed as blood biomarkers for AD to aid in early diagnosis and the development of new therapies [2, 25]. However, the ubiquitous nature of these brain-reactive autoantibodies in AD patients and healthy controls alike has led some to dismiss their usefulness as potential biomarkers of disease progression [7]. The answer to this dichotomy may be the BBB, which is found intact in healthy brains [26], becomes more permeable in old age [27], and is commonly compromised in AD brains [28]. The presence of anti-neuronal antibodies in association with BBB dysfunction could be an important contributor to AD neuropathology [28, 29].

The BBB in healthy individuals strictly controls the microenvironment of the brain by restricting the entry of blood components, including antibodies, cytokines, other soluble proteins, lymphocytes, and blood cells in general, into the brain parenchyma [29, 30]. It is well established that compromise in the cerebrovascular system plays a significant role in the initiation and progression of AD [31, 32]. This penetration of the BBB by blood components such as antibodies was shown by the detection of immunoglobulin-positive neurons in the histological study of postmortem AD brains, but not in the comparable brain region of the age-matched controls [7, 33–35]. In addition, it has been demonstrated that in human serum, brain-reactive antibodies are both numerous and ubiquitous, but in the context of BBB failure may play a role in AD pathology [7]. These findings implied that, in AD, a compromised BBB may allow brain-reactive autoantibodies in the blood that are already known as autoantigens associated with AD to gain access to the neurons within the brain tissue [7, 36]. Indeed, in one very elegant experiment with mice, the researchers [37] confirmed the presence of soluble peptides, immunoglobulins, and complement components in the blood leaking from the blood vessels and entering into the brain tissue after the induction of BBB disruption by bacterial toxin. This was shown by the influx of fluorescent-labeled Aß-42 from blood into the brain, which was not observed in the brains of healthy mice with intact BBB [38]. These findings suggest a relationship between breakdown of the BBB and the entry of soluble Aß peptides and antibodies into the brain tissue, where their association with neurons plays a role in the pathogenesis of AD [38–40].

How exactly does the penetration of these brain-reactive autoantibodies through the BBB affect the brain and the development of neurodegenerative and neuroautoimmune diseases? As Katrina Ray puts it in the March 2018 issue of Nature Collections [41], aptly titled “Gut-brain axis,” “It is becoming increasingly evident that bidirectional signalling exists between the gastrointestinal tract and the brain, often involving the gut microbiota. This relationship, commonly dubbed the gut-brain axis (or the microbiota-gut-brain axis), involves various afferent and efferent pathways such as the vagus nerve and the hypothalamic-pituitary-adrenal pathway to regulate aspects of homeostasis such as satiety and hunger, and inflammation.” As is said elsewhere in this collection of gut-brain axis articles, disruption of the gut-brain axis has been implicated in the etiopathogenesis or manifestation of a diverse range of neurodevelopmental, psychiatric, and neurodegenerative diseases, including autism spectrum disorder, depression, Alzheimer's disease, and Parkinson's disease [41]. In turn, common pathophysiological mechanisms have been associated with gastrointestinal comorbidity [41]. It is all interconnected. The gut can affect the brain, the brain can affect the gut, and they both can affect and be affected by the immune system.

What is still not completely clear is where these ubiquitous brain-reactive autoantibodies come from in the first place. Some of these antigens can be found widespread throughout the central nervous system, the peripheral nervous system, and the enteric nervous system or ENS. GFAP and claudin-5 can be found both in the BBB and the enteric nervous system, which permeates the GI tract [42–44]. The ENS consists of a mesh-like system of neurons that governs the function of the gastrointestinal tract and is capable of autonomous functions such as coordination of the reflexes [45]. The main antigen of the ENS is enteric nerve neuronal nuclear antigen (enteric nerve NNA); antibodies against this antigen are detected in patients with irritable bowel syndrome [46].

In our earlier study [47] we showed that these brain-reactive autoantibodies may originate from cross-reactive epitopes shared by A*β*P-42 with different infectious pathogens. Unpublished data from another one of our studies also indicate that these antibodies may be a result of cross-reactivity between A*β*P-42 and food antigens, and possibly from protein misfolding of A*β*P-42 by aluminum, heavy metals, and other toxic chemicals.

Consequently, we examined the immunoreactivity of monoclonal A*β*P-42 antibody with different antigens, some of which are known as autoantigens associated with AD. We needed to match this immunoreactivity with the autoantibodies that are cross-reactive with some antigens but not with others. Since the BBB seems to be compromised in AD patients, we also sought to detect antibodies against BBB components such as S100B, AQP4, claudin-5, and GFAP in their blood, and to determine if they were immunoreactive with monoclonal A*β*P-42 antibody, as this could contribute to BBB breakdown and AD neuropathology. Finally, as to the origin of these brain-reactive autoantibodies, since microbial transglutaminase (mTG) and tissue transglutaminases (tTG) have been shown by earlier studies to be involved with celiac and other autoimmune disorders [22–24], we theorized that perhaps some of these autoantibodies may arise from reactivity with mTG or cross-reactivity between enteric neuronal antigens and those that are expressed in the brain [46].

## 2. Materials and Methods

### 2.1. Antibody and Antigens

Rabbit monoclonal anti-amyloid-*β* 1-42 antibody (fibril sequence DAEFRHDSGYEVHHQKLVFFAEDVGSNKGAIIGLMVGGVVIA) was purchased from Abcam. The specificity of this antibody is shown by the fact that it reacts strongly to human A*β*42 monomers, oligomers, and fibrils, but not with human muscle fibrils. Additional information about the specificity of this antibody is provided in the Abcam package insert (ab201061) and in an article by Hatami et al. [48].

Proteins, including amyloid ß protein (AßP), tau protein, MBP, asialoganglioside GM_1_, and transglutaminase-2 (tTG-2), were purchased from Sigma-Aldrich (St Louis, MO). *β*-NGF and BDNF were purchased from Sino Biological Inc. (Wayne, PA). Different peptides, A*β*P-42, S100B, AQP4, claudin-5, GFAP, rab-5, ATP-synthase, presenilin, *α*-synuclein, enteric nerve NNA, tTG-3, mTG, glutamate-R, NMDAR, dopamine receptors 1 and 2, and GAD-65, all with purity of greater than 90%, were synthesized by Biosynthesis (Lewisville, TX).

### 2.2. Blood Samples

Sera from 47 Alzheimer's patients (Caucasian: 37, African-American: 6, and Hispanic: 4), 32 males and 15 females, ages ranging from 60 to 82 years, were purchased from Reprocell (Beltsville, MD) and Sanguine BioSciences (Valencia, CA). They were diagnosed according to the National Institute of Neurological and Communicative Disorders and Stroke and the Alzheimer's Disease and Related Disorders Association (NINCDS-ADRDA) criteria [49] with 13 having mild cognitive impairment, 12 having early AD, and 22 having moderate to late-stage AD. Sera from 47 control subjects aged 60-75 years were purchased from Innovative Research (Southfield, MI, USA). These samples were obtained from individuals who were selected as blood donors based on a modified version of the DHW v.2.0 form for screening an individual's qualifications to donate blood. Although these individuals were qualified to donate blood based on their medical history, no information was obtained on whether or not these individuals had previously suffered from or were in the process of developing autoimmune diseases. Each individual at the time of blood draw did not exhibit any health complaints. Prior to shipping, each blood sample tested negative according to FDA guidelines for hepatitis B surface antigen, antibodies to HIV, antibodies to hepatitis C, HIV-1 RNA, hepatitis C RNA, and syphilis.

### 2.3. Reaction of Anti AßP-42 with Different Neuronal Antigens

Proteins and peptides at a concentration of 1 mg/mL were diluted 1:100 in 0.1 M carbonate buffer; 100 *μ*l or 1 *μ*g of each antigen was added to a series of microtiter ELISA plate wells. After incubation for 6 hrs at room temperature (RT) and 18 hrs at 4°C, plates were washed 3 times using ELISA washer, and 200 *μ*l of 2% BSA was added to each well and incubated for 24 hrs at 4°C in order to block the nonspecific binding of the antibody to the antigen-coated wells. 100 *μ*l of monoclonal rabbit anti-A*β*P-42 diluted 1:500 in 2% BSA with 0.1 M PBS 0.05% Tween 20 was added to quadruplicate wells of different ELISA plates coated with BSA only or various neuronal or other antigens. After washing 5 times with 0.1 M PBS 0.05% Tween 20, 100 *μ*l of alkaline phosphatase-labeled anti-rabbit IgG at a dilution of 1:600 was then added to all wells and incubated again for 1 hour at room temperature. The enzyme reaction was started by adding 100 *μ*L of paranitrophenyl phosphate at a concentration of 1 mg/mL in diethanolamine buffer containing 1 mM MgCl_2_ and sodium azide at a pH of 9.8. The reaction was stopped 45 minutes later with 50 *μ*L of 1 N NaOH, and the samples were read by an ELISA reader; the optical densities were measured at 405 nM.

To determine the specificity of rabbit monoclonal anti-A*β*P-42 binding to the neuronal antigens, the rabbit monoclonal antibody was replaced with the same dilution of nonimmunized rabbit serum and added to quadruplicate wells. Furthermore, the anti-A*β*P-42 and other reagents were added to 4 wells coated with human serum albumin (HSA) and 4 wells coated with 2% BSA alone; these were then used as negative controls. After the addition of other reagents to these control wells, the ODs were measured and their mean was subtracted from the mean OD of all other reactions.

### 2.4. ELISA Determination of Neuronal Antibody in Sera from Controls and Patients with AD

For the measurement of IgG antibody against AßP-42 and other antigens in the sera of patients with AD in comparison with controls, the sera were diluted 1:100, and 100 *μ*l was added to quadruplicate wells of a microtiter plate coated with AßP-42 and 22 different antigens. After incubation for one hour at 24°C, plates were washed 3 times with 0.1 M PBS Tween 20, and 100 *μ*l of alkaline phosphatase-labeled goat anti-human IgG F(ab^1^)_2_ fraction at a dilution of 1:600 was added to all wells. The plates were incubated again for one hour at RT. After washing 5 times with TBS-Tween buffer, the enzyme reaction was started with the addition of 100 *μ*l paranitrophenyl phosphate in 0.1 mL diethanolamine buffer 1 mg/mL containing 1 mM MgCl_2_ and sodium azide pH 9.8. The reaction was stopped 45 mins later by adding 50 *μ*l of 1 N NaOH. To detect nonspecific binding, several wells containing all reagents except human serum, or wells coated with HSA or rabbit serum, were used as controls. The ODs for all these negative control wells were <0.2, and for positive control wells it went as high as 3.8.

### 2.5. Binding of Serially Diluted Anti-AßP-42 with Various Neural Antigens

For the demonstration of the specificity of anti-AßP-42 antibody binding to different neural antigens, four different strips of microtiter plate, each containing 8 wells, one strip coated with A*β*P-42, the second with presenilin, the third with *β*-NGF, and the fourth with tau protein, were used. These four antigens were chosen as being representative of all the antigens that showed immune reactivity to anti-A*β*P-42 ranging from highly positive to very highly positive. Anti-A*β*P serially diluted from 1:500 to 1:64,000 was then added to the appropriate wells of the microtiter plate. After incubation, washing, and the addition of the secondary antibody, plus all other steps for the completion of the ELISA assays, the ODs were recorded at 405 nM.

### 2.6. Inhibition of Anti-AßP Antibody Binding to Neural Antigen-Coated Plates with the Same Antigens in Liquid Phase

100 *μ*l of diluents was added to all wells of four different rows of microtiter plates coated with either AßP-42, presenilin, tau protein, or BDNF. 20 *μ*l of 0.1 M PBS was added to the first well of each row; to the additional antigen-coated wells, 20 *μ*l of PBS containing 1.25-80 *μ*g AßP-42, presenilin, tau protein, or BDNF was added, respectively. After the addition of the secondary antibody and completion of all ELISA steps, the ODs were recorded at 405 nM.

### 2.7. Statistical Analysis

Statistical analysis was performed to study the linear relationship between the presence of anti-AßP-42 antibody and the antibody levels against 22 different antigens in healthy controls and in AD patients, resulting in a significant* p* value of* p*≤ 0.001. The determination of the presence of statistically significant correlative relationship was conducted with Pearson's correlation coefficients. A Bonferroni adjustment to adjust for multiple comparisons was used in the analysis to avoid a false discovery rate when testing for multiple comparisons, resulting in a significant* p* value of 0.002. STATA software package was used to perform all inferential analysis.

## 3. Results

### 3.1. The Immune Reactivity of Anti-Aß-42 Peptide with 22 Neuronal and Other Tissue Antigens

**Figure 1:**
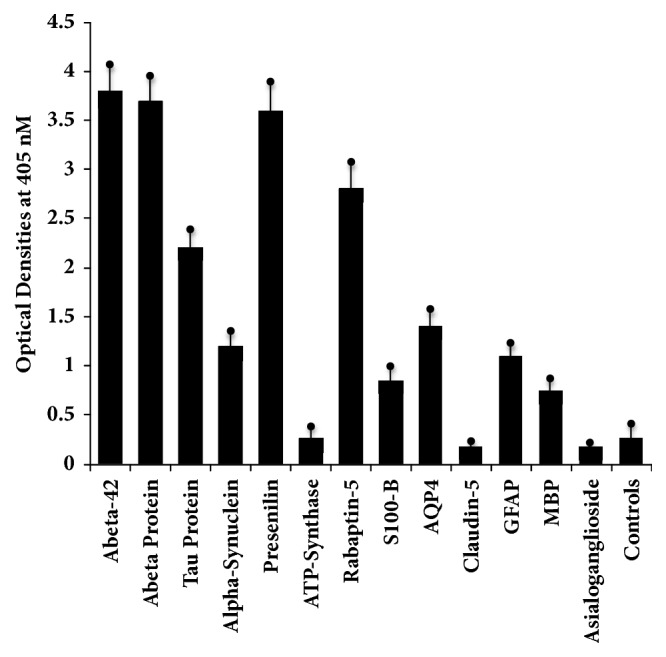
Reaction of monoclonal antibody to A*β*42 with A*β*42 peptide and different proteins directly or indirectly involved with AD. The mean ± 3SD of 12 determinations for each antigen is shown. Compared to the monoclonal antibody's reaction with amyloid-*β*-42 as positive control and HSA or unimmunized rabbit serum as negative control, the reaction of this antibody with ATP-synthase, claudin-5, and asialoganglioside GM_1_ is nonreactive, with S100-B, GFAP, and MBP is low or weakly positive, with *α*-synuclein and AQP4 is moderate, with tau protein and rabaptin-5 is highly positive, and with A*β* protein and presenilin is very highly positive. 0-0.27 OD: nonreactive, 0.271-0.50: equivocal, 0.51-1.2: low positive, 1.21-2.0: moderately positive, 2.01-3.0: highly positive, and >3.0: very highly positive.

**Figure 2:**
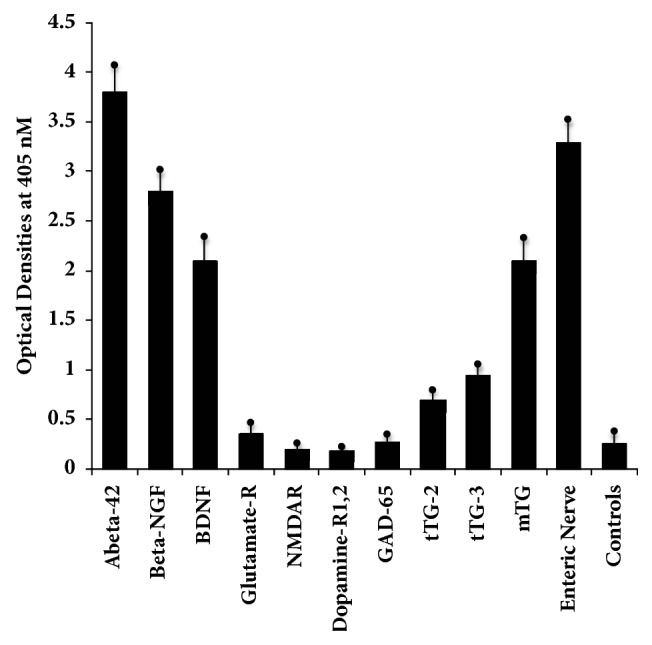
Reaction of rabbit monoclonal antibody to A*β*42 with A*β*42 peptide and different proteins in the brain and in the gut directly or indirectly involved with AD. The mean ± 3SD of 12 determinations for each antigen is shown. Compared to the monoclonal antibody's reaction with amyloid-*β*-42 as positive control and HSA or unimmunized rabbit serum as negative control, the reaction of this antibody with NMDAR, dopamine-R1 and R2, and GAD-65 is nonreactive, with glutamate-R is equivocal, with tTG-2 and tTG-3 is low or weakly positive, with *β*-NGF, BDNF, and mTG is highly positive, and with enteric nerve NNA is very highly positive. 0-0.27 OD: nonreactive, 0.271-0.50: equivocal, 0.51-1.2: low positive, 1.21-2.0: moderately positive, 2.01-3.0: highly positive, and >3.0: very highly positive.

We measured the immune reactivity of anti-AßP-42 peptide with neuronal and other tissue antigens that may play a role in neurodegenerative disorders, particularly AD. For simplification of the antibody reactivity results, we used the following key: 0-0.27 OD: nonreactive, 0.271-0.50: equivocal, 0.51-1.2: low positive, 1.21-2.0: moderately positive, 2.01-3.0: highly positive, and >3.0: very highly positive. Using ELISA methodology for demonstration of this immune reaction, we first found that the strongest reaction was observed between anti-Aß-42 and peptide 1-42 itself with OD of 3.8 or very highly positive, which is very close to the maximum detection limits of the assay (4.0). In relation to the other neuronal proteins, the reaction to this monoclonal anti-AßP-42 antibody was very highly positive with Aß protein, presenilin, and enteric nerve NNA. The same antibody had a highly positive reaction with tau protein, BDNF, *β*-NGF, rab-5, and mTG, an enzyme widely used as a food additive. The antibody was moderately positive with *α*-synuclein and AQP4, and low or weakly positive with S100B, MBP, GFAP, tTG-3, and tTG-2 (Figures 1 and 2). The OD for glutamate-R was 0.35, which is equivocal. The antibody did not react with ATP-synthase, asialoganglioside GM_1_, claudin-5, NMDAR, dopamine receptors I and 2, and GAD-65. The ELISA ODs for all these reactions were within 3SD above the mean of control values or 0.27. The strength of immunoreactivity of AßP-42 antibody and its reactivity with 22 different antigens relative to AßP-42 binding to AßP-42 peptide as 100% is shown in Table 1.

### 3.2. Demonstration of Specificity of Monoclonal Anti-Aß-42 Antibody Binding to Different Neural Antigens

**Figure 3:**
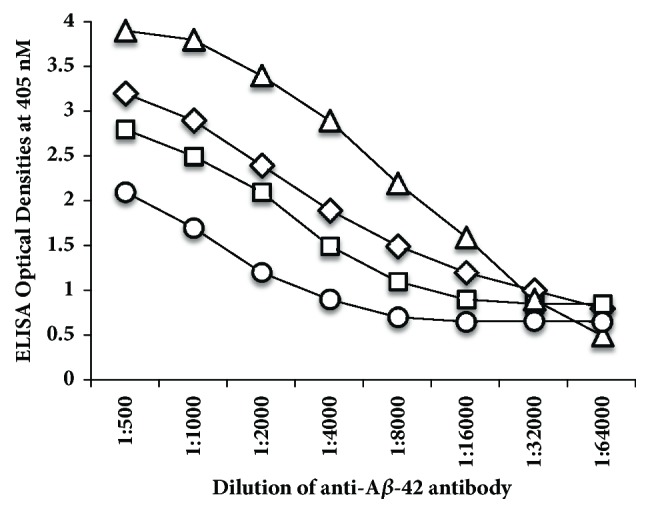
Reaction of serially diluted anti-A*β*P-42 with A*β*P-42 △, presenilin ⋄, *β*-NGF □, and tau protein ○ coated ELISA microwells. These four antigens were chosen as being representative of all the antigens that showed immune reactivity to anti-A*β*P-42 ranging from highly positive to very highly positive. In proportion to the dilution, a significant decline in the reaction of anti-amyloid-*β* peptide with all 4 antigens was observed.

The specificity of the anti-Aß-42 binding to different neural antigens was confirmed by serial dilution and inhibition studies. As shown in Figure 3, similar to the decline in AßP-42 antibody binding to AßP-42 in proportion to the dilution, the binding of this antibody to the same concentration of presenilin, ß-NGF, and tau protein declined significantly. For example, anti-AßP-42 at a dilution of 1:500 with presenilin gave an OD of 3.2, a dilution of 1:8000 resulted in an OD of 1.5, and a dilution of 1:64000 gave an OD of 0.8. Similar results were obtained with serially diluted antibody and its reaction with ß-NGF and tau protein (Figure 3).

**Figure 4:**
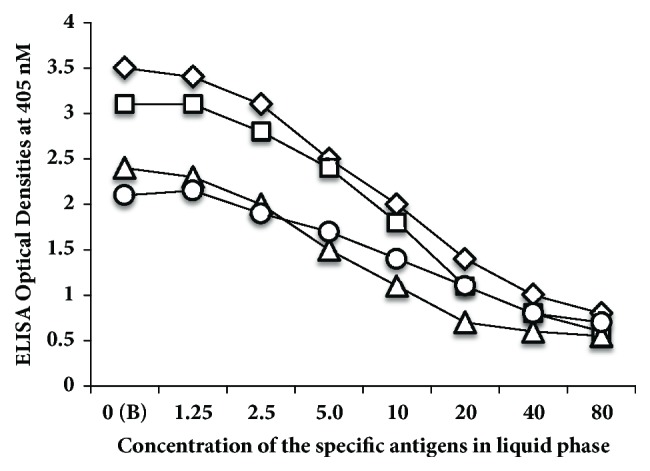
Inhibition of anti-A*β*P-42 binding to A*β*P-42 ⋄, presenilin □, tau protein △, and BDNF ○ coated ELISA microwells with different concentrations from 0-80 *μ*g of the same peptides or proteins in the liquid phase. These four antigens were chosen as being representative of all the antigens that showed immune reactivity to anti-A*β*P-42 ranging from highly positive to very highly positive. The higher the concentration of antigens used as inhibitor, the lower the reaction of anti-A*β*P-42 binding to different antigens.

To further demonstrate the specificity of these reactions between AßP-42 antibody and neural antigens, different amounts of neural antigens (inhibitors) in concentrations of 1.25-80 *μ*g or controls (no inhibitors) were added in the liquid phase of the ELISA plates that were coated with optimal concentrations of presenilin, tau protein, and BDNF (Figure 4). Compared to the control shown in Figure 4 as B or blank, the addition of neural antigens to the liquid phase of the assay resulted in significant inhibition in AßP-42 antibody binding to presenilin, tau protein, and BDNF in proportion to the concentrations of the inhibitors. Compared to the blank or control with nonspecific protein (HSA), this inhibition of antibody-antigen reaction was more obvious when higher concentrations of the neural antigens were added to the liquid phase (Figure 4).

### 3.3. Detection of Anti-Neuronal Antibodies in Blood of Controls and Patients with AD

**Figure 5:**
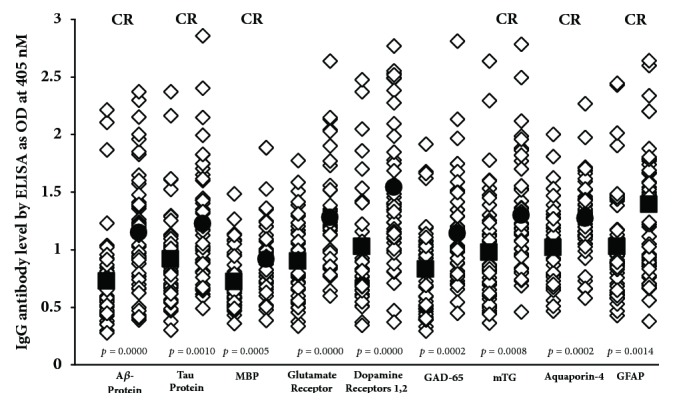
IgG antibodies against various proteins and peptides that are directly or indirectly involved in AD and may be associated with AD as autoantigens, with significant* p* values of 0.001 or less. ■: mean of controls, ●: mean of AD patients, and CR: cross-reactive with A*β*P-42.

**Figure 6:**
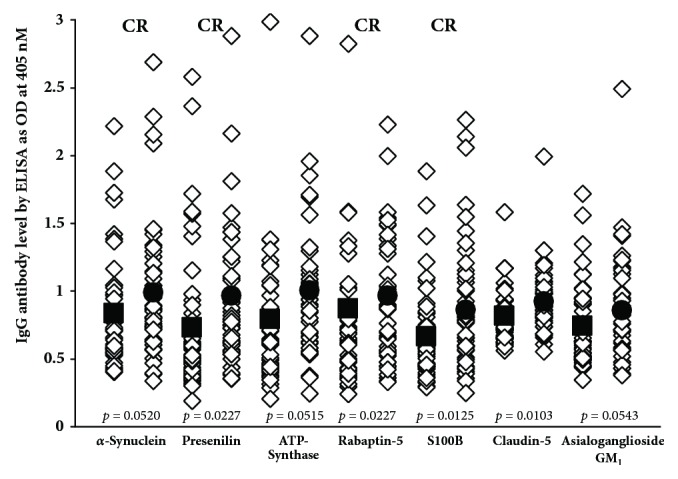
IgG antibodies against various proteins and peptides that are directly or indirectly involved in AD and may be associated with AD as autoantigens, with insignificant* p* values. ■: mean of controls, ●: mean of AD patients, and CR: cross-reactive with A*β*P-42.

**Figure 7:**
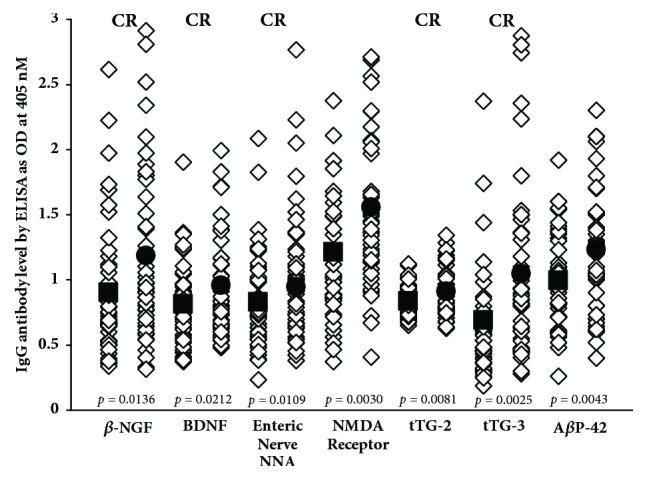
IgG antibodies against various proteins and peptides that are directly or indirectly involved in AD and may be associated with AD as autoantigens, with insignificant* p* values. ■: mean of controls, ●: mean of AD patients, and CR: cross-reactive with A*β*P-42.

Data presented in scattergrams (Figures 5–7) show that a significant variation in the level of antibodies expressed by ELISA ODs exist both in nondemented controls and AD sera.  Figure 5 shows that the nine antigens with significant* p* values (*p* ≤ 0.001) are A*β* protein, tau protein, MBP, glutamate-R, dopamine receptors 1 and 2, GAD-65, mTG, AQP4, and GFAP. We calculated the percentage of elevation of these antibodies at 2SD over the mean of the controls in both sera from controls (6-11%) and patients with AD (24-37%), which can be seen in Table 2. It should be noted that, out of these nine antigens, six reacted strongly to monoclonal anti-A*β*P-42. Figures 6 and 7 show A*β*P-42 and thirteen other proteins and peptides that had insignificant* p* values. Table 2 also shows the percentages of elevation for A*β*P-42 and these other proteins and peptides in both controls (4-11%) and AD patients (6-31%). Examination of Figures 6 and 7 shows that a significant number of controls as well as Alzheimer's patients exhibit elevations in the levels of antibodies above the mean. Comparison of the two groups therefore resulted in statistically insignificant* p* values. It is interesting to note as well that, of the thirteen antibody measurements, nine antigens also reacted strongly to monoclonal anti-A*β*P-42, which, despite their* p* values, makes their presence in the blood significant indeed (see Figures 6 and 7).

The percentage of these autoantibodies in AD patients and healthy controls is shown in Table 2.

### 3.4. Correlation Coefficients between IgG Anti-Aß-42 Antibody and Antibodies against Tested Proteins or Peptides

The correlation coefficient between blood levels of IgG anti-AßP-42 with 22 different antibodies against neural or associated antigens or peptides was performed using Pearson's correlation coefficients. Since IgG antibodies against AßP-42 are detected in the blood of healthy subjects or AD patients, we sought to determine if IgG antibody could also be detected against the other 22 tested proteins and peptides. The correlation coefficients were found to be between 0.20 and 0.98 (Table 3). This means that if IgG antibody is detected against AßP-42 in the blood of controls or AD patients, the probability of detecting high IgG antibody against amyloid ß protein, tau protein, *α*-synuclein, or GFAP in the same individual is more than 90%. With enteric neuronal antigen, *β*-NGF, and BDNF, the correlation was more than 80%. For the other antibodies the correlation ranged from 20% to 80%. To correct for multiple comparisons we used a Bonferroni correction, dividing the alpha by .05/22, giving a significant***p value of 0.002.***

## 4. Discussion

In this study, the immune cross-reactivity between anti-AßP-42 antibodies with a variety of brain-associated proteins and peptides was examined using monoclonal anti-AßP-42 and ELISA methodology. Although the exact mechanism of this cross-immunoreactivity between AßP-42 and so many brain-associated antigens shown in this study is not clear, a significant amino acid sequence homology between A*β*P-42, tau protein, and NGF has been shown by Carter [50]. Carter postulated that AßP antibodies as well as tau protein and NGF antibodies observed in AD may well be autoantibodies to pathogens, due to their homology with human autoantigens [50–52]. Other studies [13, 53, 54] have also shown a significant homology between plants and bacterial aquaporin with human AQP4 that is expressed in the astrocytic foot processes. Antibodies against plant AQP4 from tomato, corn, soy, spinach, and human AQP4 have been detected in patients with MS and neuromyelitis optica [13, 54]. Carter cited other studies which found that tau or NGF antibodies promote amyloid-ß deposition, neurofibrillary tangles, and neuronal cell destruction, whose process is dictated by sequence homology between pathogens and human proteins [55, 56]. Rosenmann et al. found that vaccination with tau proteins induced histopathologic features of Alzheimer's disease and tauopathies, indicated by the presence of neurofibrillary tangle-like structures, axonal damage, and gliosis [56]. Simply put, autoantibodies to these endogenous proteins (NGF or tau) can produce Alzheimer's-like pathology.

Previous studies have shown IgG positive neurons in the brains of AD patients in the context of BBB compromise [27, 34, 38]. Our results provide evidence that sera from AD patients contain autoantibodies that react strongly with some proteins involved in the BBB and other proteins that are recognized as autoantigens in AD. Some of these antibodies may arise due to immunoreactivity against mTG and/or enteric neuronal antigens, since our testing showed that monoclonal antibody made against A*β*P-42 reacted very strongly with these antigens. We also found antibodies against AßP-42, tau protein, ß-NGF, and many other associated proteins including claudin-5, S100B, GFAP, and AQP4 not only in patients with AD but in a significant number of sera from healthy subjects. Overall, this detection of 23 brain-reactive and other associated antibodies in the blood of healthy subjects is supported by an earlier report by Nagele et al. [35] that dealt with a smaller number of antigens. It was suggested that, in the BBB, brain-reactive antibodies are ubiquitous and that defects in BBB permeability allow these antibodies and blood-borne components to access the brain interstitium, where they manage to bind to neuronal cells and enhance intraneuronal deposition of AßP-42 in the brain, which may contribute to the immunopathology of AD [2, 27–34, 36]. In the same way, Gebhard et al. [57] showed that, in a disease state, streptococcal antibodies were able to cross the BBB and interact with tissues of the basal ganglia. This may explain why these brain-reactive autoantibodies can safely stay in the circulatory system of normal individuals with intact BBBs, but in patients with compromised BBBs may lead to neurodegenerative disorders such as AD. Thus, in the context of cross-reactivity between AßP-42 antibodies, BBB proteins such as S100B, AQP4, claudin-5, and GFAP, and many other brain-associated antigens, BBB compromise may be an important risk factor in the intraneuronal deposition of AßP-42 and in the initiation and/or progression of neurodegenerative diseases, including AD. If a compromised BBB were to allow mTG, *β*-NGF, BDNF, enteric nerve NNA, and other neuronal antibodies to reach the brain, they may react with AßP-42 and possibly other proteins involved in AD. Therefore, protecting the BBB from the entry of unwanted molecules that may induce inflammatory responses in the brain should be one major strategy for the prevention of AD and other neurodegenerative disorders. In addition to the importance of BBB compromise, it is crucial to find the origin of the AßP-42 cross-reactive antibodies that are detected in the blood of healthy subjects and AD patients. In our earlier studies [47] we showed several pathogens and antigens such as LPS, bacterial cytolethal distending toxin, and others that may contribute to the presence of AßP-42 cross-reactive antibodies. The identification of environmental triggers that cross-react with AßP-42 antibodies and contribute to amyloidogenesis may help clinicians to develop treatment protocols involving the removal of these triggers. Many attempts have been made to stop or reverse cognitive decline in AD. There are FDA-approved medications such as Donepezil, Rivastigmine, Galantamine, and Memantine [58–60]. It should be noted that the FDA-approved medications listed here only treat the symptoms of AD and do not stop or reverse cognitive decline. No treatment has yet been definitively qualified to be a disease-modifying treatment. There are also alternative solutions such as lifestyle modification as a novel therapeutic program [61], phytochemical ginkgolide [62], berberine from barberry [63], synthesized curcumin derivatives [64], and Resveratrol [65]. These interventions deal with gut microbiome-related changes and microbiome-derived molecules that play a significant role in the induction of BBB permeability and the leak of cross-reactive antibodies and neurotoxic molecules into the cerebral vasculature and into the brain [66]. Therefore, in addition to blood level of brain cross-reactive antibodies, the internal source of antigenic stimuli that activate the innate and adaptive immune responses is equally important [66–69].

We believe that our results provide credible evidence for a mechanism in which environmental factors and the production of A*β*P-42 cross-reactive antibodies in conjunction with a compromised BBB all combine to contribute to AD pathogenesis. Among these antigens whose antibodies are reactive with A*β*P-42, we propose that special attention should be given to mTG, *β*-NGF, BDNF, component BBB antigens, and enteric neuronal antigens. We theorize that consumption of mTG and related food proteins may result in the production of brain-immuno-cross-reactive autoantibodies. This can happen even in subjects who would be classified as nominally healthy, that is to say, not having Alzheimer's disease or other neurodegenerative disorders. However, in individuals with compromised blood-brain barriers, these A*β*P-42 cross-reactive autoantibodies shown in Figures 5–7 may be able to reach the interstitium of the brain, where they can react with the key neural protein A*β*P-42. The immunoreactivity of these cross-reactive antibodies with A*β*P-42 contributes towards the deposition of A*β*P-42 and the formation of amyloid plaques that are the hallmark of AD. The antigenic similarity or homology between pathogens, food antigens such as mTG, and tissue antigens with AßP-42 and other neuronal antigens may be the mechanism by which these brain-reactive autoantibodies attack the brain's own cells. This brings us to *β*-NGF and BDNF, factors that are so important in neuronal regeneration [70, 71]. *β*-NGF supports the survival and growth of neural cells, regulates cell growth, promotes differentiation of neurons, and aids in neuron migration [72]. BDNF plays a vital role in the growth, development, maintenance, and functioning of several neuronal systems [73]. We believe that antibodies produced against *β*-NGF and BDNF due to their cross-reactivity with A*β*P-42 not only enhance the process of amyloidogenesis but may prevent the normal healing and replacement of these nerve cells. All of these factors and processes can combine, resulting in neurodegeneration and the neuropathology of AD and other neurological disorders.

By using the methods shown in this study to identify the triggers that induce the production of A*β*P-42 cross-reactive antibodies, it will be possible to remove triggers such as mTG and develop therapeutic protocols including proper diets and supplements that will help repair the body's compromised barriers, restore the immune system to proper functioning, and hopefully improve the AD patient's quality of life.

## Figures and Tables

**Table 1:** Comparison of immunoreactivity of anti-A*β*P-42 antibody with different neuronal or associated antigens using reactivity with AßP-42 peptide as 100%.

**Variables**	**Percentage of reactivity**	**Strength of reactivity w/ monoclonal A** **β** **P-42**
AßP-42 peptide	100%	Very highly positive
Amyloid *β* protein	97%	Very highly positive
Tau protein	58%	Highly positive
*α*-synuclein	32%	Moderately positive
Presenilin	95%	Very highly positive
Adenosine triphosphate synthase	7%	Non-reactive
Rabaptin-5	74%	Highly positive
S100B	22%	Low positive
Aquaporin-4	37%	Moderately positive
Claudin-5	5%	Non-reactive
Glial fibrillary acidic protein	29%	Low positive
Myelin basic protein	20%	Low positive
Asialoganglioside GM_1_	5%	Non-reactive
*β*-nerve growth factor	74%	Highly positive
Brain-derived neurotrophic factor	55%	Highly positive
Glutamate receptor	10%	Equivocal
*N*-methyl-D-aspartate receptor	6%	Nonreactive
Dopamine receptors 1, 2	5%	Nonreactive
Glutamic acid decarboxylase 65	7%	Nonreactive
Transglutaminase-2	18%	Low positive
Transglutaminase-3	25%	Low positive
Microbial transglutaminase	55%	Highly positive
Enteric nerve neuronal nuclear antigen	87%	Very highly positive

0-0.27 OD: nonreactive, 0.271-0.50: equivocal, 0.51-1.2: low positive, 1.21-2.0: moderately positive, 2.01-3.0: highly positive, and >3.0: very highly positive.

**Table 2:** Percentage of elevation of autoantibodies in controls and AD patients at 2SD above the mean of controls.

**Variables**	%** Controls**	%** AD Patients**
AßP-42 peptide	4%	22%
Amyloid *β* protein	8%	37%
Tau protein	11%	26%
*α*-synuclein	8%	13%
Presenilin	13%	17%
Adenosine triphosphate synthase	8%	17%
Rabaptin-5	6%	6%
S100B	8%	22%
Aquaporin-4	8%	22%
Claudin-5	11%	15%
Glial fibrillary acidic protein	8%	28%
Myelin basic protein	6%	20%
Asialoganglioside GM_1_	8%	15%
*β*-nerve growth factor	11%	26%
Brain-derived neurotrophic factor	6%	17%
Glutamate receptor	6%	26%
*N*-methyl-D-aspartate receptor	8%	26%
Dopamine receptors 1, 2	11%	28%
Glutamic acid decarboxylase 65	8%	22%
Transglutaminase-2	6%	24%
Transglutaminase-3	8%	31%
Microbial transglutaminase	6%	24%
Enteric nerve neuronal nuclear antigen	6%	24%

**Table 3:** Correlations between IgG anti-A*β*P-42 antibody with IgG against other neuronal or associated proteins.

**Variables**	**P values**	**Correlation coefficients**
Amyloid *β* protein	0.0001	0.9680
Tau protein	0.0001	0.9497
*α*-synuclein	0.0001	0.9760
Presenilin	0.0001	0.7964
Adenosine triphosphate synthase	0.0001	0.4597
Rabaptin-5	0.0001	0.5892
S100B	0.0001	0.6892
Aquaporin-4	0.0001	0.8286
Claudin-5	0.0053	0.3361
Glial fibrillary acidic protein	0.0001	0.9286
Myelin basic protein	0.0001	0.4911
Asialoganglioside GM_1_	0.0146	0.2631
*β*-nerve growth factor	0.0001	0.8971
Brain-derived neurotrophic factor	0.0001	0.8743
Glutamate-Receptor	0.0112	0.2871
*N*-methyl-D-aspartate receptor	0.0129	0.2541
Dopamine receptors 1, 2	0.0616	0.1982
Glutamic acid decarboxylase 65	0.0173	0.2463
Transglutaminase-2	0.0001	0.4981
Transglutaminase-3	0.0001	0.6137
Microbial transglutaminase	0.0001	0.7792
Enteric neuronal antigen	0.0001	0.8963

We tested 22 variables for correlation and made a correction to the* p* value for multiple comparisons to avoid a false discovery rate, since, statistically, with an alpha value of 0.05, 1 out of 20 correlations would be a false positive. We used a Bonferroni correction by dividing the alpha by .05/22, resulting in a significant **p value of 0.002.**

## Data Availability

The data used to support the findings of this study are available from the corresponding author upon request.

## Additional Points

*Limitations.* The authors acknowledge that the study is somewhat limited by the relatively small number of samples (47 Alzheimer's patients, 47 healthy controls). It was also limited by not having complete information about whether the control samples had previously suffered from an autoimmune disease or were actually in the preliminary silent stages of an autoimmune disorder. They acknowledge as well that the fact that funding for this study was provided by the authors is also a limitation.

## References

[1] Itzhaki R. F., Lathe R., Balin B. J. (2016). Microbes and Alzheimer’s Disease. *Journal of Alzheimer's Disease*.

[2] Colasanti T., Barbati C., Rosano G., Malorni W., Ortona E. (2010). Autoantibodies in patients with Alzheimer's disease: pathogenetic role and potential use as biomarkers of disease progression. *Autoimmunity Reviews*.

[3] Bourgade K., Le Page A., Bocti C. (2016). Protective Effect of Amyloid-*β* Peptides Against Herpes Simplex Virus-1 Infection in a Neuronal Cell Culture Model. *Journal of Alzheimer's Disease*.

[4] Bourgade K., Garneau H., Giroux G. (2014). *β*-Amyloid peptides display protective activity against the human Alzheimer’s disease-associated herpes simplex virus-1. *Biogerontology*.

[5] Kumar D. K. V., Choi H. S., Washicosky K. J. (2016). Amyloid-*β* peptide protects against microbial infection in mouse and worm models of Alzheimer's disease. *Science Translational Medicine*.

[6] Ludwig R. J., Vanhoorelbeke K., Leypoldt F. (2017). Mechanisms of Autoantibody-Induced Pathology. *Frontiers in Immunology*.

[7] Levin E. C., Acharya N. K., Han M. (2010). Brain-reactive autoantibodies are nearly ubiquitous in human sera and may be linked to pathology in the context of blood-brain barrier breakdown. *Brain Research*.

[8] Maftei M., Thurm F., Schnack C. (2013). Increased Levels of Antigen-Bound *β*-Amyloid Autoantibodies in Serum and Cerebrospinal Fluid of Alzheimer’s Disease Patients. *PLoS ONE*.

[9] Kolarova M., García-Sierra F., Bartos A., Ricny J., Ripova D. (2012). Structure and pathology of tau protein in Alzheimer disease. *International Journal of Alzheimer's Disease*.

[10] Deber C. M., Reynolds S. J. (1991). Central nervous system myelin: structure, function, and pathology. *Clinical Biochemistry*.

[11] D'Aversa T. G., Eugenin E. A., Lopez L., Berman J. W. (2013). Myelin basic protein induces inflammatory mediators from primary human endothelial cells and blood-brain barrier disruption: Implications for the pathogenesis of multiple sclerosis. *Neuropathology and Applied Neurobiology*.

[12] Vacirca D., Delunardo F., Matarrese P. (2012). Autoantibodies to the adenosine triphosphate synthase play a pathogenetic role in Alzheimer's disease. *Neurobiology of Aging*.

[13] Vaishnav R. A., Liu R., Chapman J. (2013). Aquaporin 4 molecular mimicry and implications for neuromyelitis optica. *Journal of Neuroimmunology*.

[14] Mecocci P., Parnetti L., Romano G. (1995). Serum anti-GFAP and anti-S100 autoantibodies in brain aging, Alzheimer's disease and vascular dementia. *Journal of Neuroimmunology*.

[15] Cox C. J., Sharma M., Leckman J. F. (2013). Brain Human Monoclonal Autoantibody from Sydenham Chorea Targets Dopaminergic Neurons in Transgenic Mice and Signals Dopamine D2 Receptor: Implications in Human Disease. *The Journal of Immunology*.

[16] Liu C., Zhu J., Zheng X., Ma C., Wang X. (2017). Anti-N-Methyl-D-aspartate Receptor Encephalitis: A Severe, Potentially Reversible Autoimmune Encephalitis. *Mediators of Inflammation*.

[17] McKeon A., Tracy J. A. (2017). GAD65 neurological autoimmunity. *Muscle & Nerve*.

[18] Mitoma H., Manto M., Hampe C. S. (2017). Immune-mediated cerebellar ataxias: from bench to bedside. *Cerebellum & Ataxias*.

[19] Thomas H., Beck K., Adamczyk M. (2013). Transglutaminase 6: A protein associated with central nervous system development and motor function. *Amino Acids*.

[20] Griffin M., Casadio R., Bergamini C. M. (2002). Transglutaminases: nature's biological glues. *Biochemical Journal*.

[21] Hadjivassiliou M., Aeschlimann P., Strigun A., Sanders D. S., Woodroofe N., Aeschlimann D. (2008). Autoantibodies in gluten ataxia recognize a novel neuronal transglutaminase. *Annals of Neurology*.

[22] Zone J. J., Schmidt L. A., Taylor T. B. (2011). Dermatitis Herpetiformis Sera or Goat Anti–Transglutaminase-3 Transferred to Human Skin-Grafted Mice Mimics Dermatitis Herpetiformis Immunopathology. *The Journal of Immunology*.

[23] Lerner A., Matthias T. (2016). Don't forget the exogenous microbial transglutaminases: It is immunogenic and potentially pathogenic. *AIMS Biophysics*.

[24] Lerner A., Matthias T. (2015). Food Industrial Microbial Transglutaminase in Celiac Disease: Treat or Trick. *International Journal of Celiac Disease*.

[25] Chintamaneni M., Bhaskar M. (2012). Biomarkers in Alzheimer's Disease: A Review. *ISRN Pharmacology*.

[26] Bowman G. L., Kaye J. A., Moore M., Waichunas D., Carlson N. E., Quinn J. F. (2007). Blood-brain barrier impairment in Alzheimer disease: Stability and functional significance. *Neurology*.

[27] Marques F., Sousa J. C., Sousa N., Palha J. A. (2013). Blood-brain-barriers in aging and in Alzheimer's disease. *Molecular Neurodegeneration*.

[28] Zipser B. D., Johanson C. E., Gonzalez L. (2007). Microvascular injury and blood-brain barrier leakage in Alzheimer's disease. *Neurobiology of Aging*.

[29] Deane R., Zlokovic B. V. (2007). Role of the blood-brain barrier in the pathogenesis of Alzheimer's disease. *Current Alzheimer Research*.

[30] Hawkins B. T., Davis T. P. (2005). The blood-brain barrier/neurovascular unit in health and disease. *Pharmacological Reviews*.

[31] Kalaria R. N. (1999). The blood-brain barrier and cerebrovascular pathology in Alzheimer's disease. *Annals of the New York Academy of Sciences*.

[32] Kalaria R. N. (2003). Vascular factors in Alzheimer's disease. *International Psychogeriatrics*.

[33] Bouras C., Riederer B. M., Kövari E., Hof P. R., Giannakopoulos P. (2005). Humoral immunity in brain aging and Alzheimer's disease. *Brain Research Reviews*.

[34] Stein T. D., Fedynyshyn J. P., Kalil R. E. (2002). Circulating autoantibodies recognize and bind dying neurons following injury to the brain. *Journal of Neuropathology & Experimental Neurology*.

[35] Nagele R. G., Clifford P. M., Siu G. (2011). Brain-reactive autoantibodies prevalent in human sera increase intraneuronal amyloid-*β* 1–24 deposition. *Journal of Alzheimer's Disease*.

[36] Clifford P. M., Zarrabi S., Siu G. (2007). A*β* peptides can enter the brain through a defective blood-brain barrier and bind selectively to neurons. *Brain Research*.

[37] D'Andrea M. R. (2003). Evidence linking neuronal cell death to autoimmunity in Alzheimer's disease. *Brain Research*.

[38] DAndrea M. R. (2014). *Bursting neuron and fading memories: An alternative hypothesis of the pathogenesis of Alzheimer’s disease. Chapter 14: The BBB and BRB in AD*.

[39] Mackic J. B., Bading J., Ghiso J. (2002). Circulating amyloid-*β* peptide crosses the blood-brain barrier in aged monkeys and contributes to Alzheimer's disease lesions. *Vascular Pharmacology*.

[40] Nagele R. G., D'Andrea M. R., Lee H., Venkataraman V., Wang H.-Y. (2003). Astrocytes accumulate A*β*42 and give rise to astrocytic amyloid plaques in Alzheimer disease brains. *Brain Research*.

[41] Ray K. Nature Collections: Gut-Brain Axis.

[42] Hao M. M., Capoccia E., Cirillo C., Boesmans W., Vanden Berghe P. (2017). Arundic Acid Prevents Developmental Upregulation of S100B Expression and Inhibits Enteric Glial Development. *Frontiers in Cellular Neuroscience*.

[43] Ochoa-Cortes F., Turco F., Linan-Rico A. (2016). Enteric Glial Cells: A New Frontier in Neurogastroenterology and Clinical Target for Inflammatory Bowel Diseases. *Inflammatory Bowel Diseases*.

[44] Lu Z., Ding L., Lu Q., Chen Y. (2014). Claudins in intestines: distribution and functional significance in health and diseases. *Tissue Barriers*.

[45] Furness J. B. (2008). The enteric nervous system: normal functions and enteric neuropathies. *Neurogastroenterology & Motility*.

[46] Wood J. D., Liu S., Drossman D. A., Ringel Y., Whitehead W. E. (2012). Anti-enteric neuronal antibodies and the irritable bowel syndrome. *Journal of Neurogastroenterology and Motility*.

[47] Vojdani A., Vojdani E., Saidara E., Kharrazian D. (2018). Reaction of Amyloid-*β* Peptide Antibody with Different Infectious Agents Involved in Alzheimer’s Disease. *Journal of Alzheimer's Disease*.

[48] Hatami A., Albay R., Monjazeb S., Milton S., Glabe C. (2014). Monoclonal antibodies against A*β*42 fibrils distinguish multiple aggregation state polymorphisms *in vitro* and in Alzheimer disease brain. *The Journal of Biological Chemistry*.

[49] Jack C. R., Albert M. S., Knopman D. S. (2011). The diagnosis of dementia due to Alzheimer’s disease: Recommendations from the National Institute on Aging-Alzheimer’s Association workgroups on diagnostic guidelines for Alzheimer’s disease. Alzheimer’s & dementia. *the journal of the Alzheimer’s Association*.

[50] Carter C. (2011). Alzheimer's disease: APP, gamma secretase, APOE, CLU, CR1, PICALM, ABCA7, BIN1, CD2AP, CD33, EPHA1, and MS4A2, and their relationships with herpes simplex, C. Pneumoniae, other suspect pathogens, and the immune system. *International Journal of Alzheimer's Disease*.

[51] Carter C. J. (2010). Alzheimer's Disease: A Pathogenetic Autoimmune Disorder Caused by Herpes Simplex in a Gene-Dependent Manner. *International Journal of Alzheimer's Disease*.

[52] Carter C. J. (2017). Genetic, Transcriptome, Proteomic, and Epidemiological Evidence for Blood-Brain Barrier Disruption and Polymicrobial Brain Invasion as Determinant Factors in Alzheimer’s Disease. *Journal of Alzheimer's Disease Reports*.

[53] Ren Z., Wang Y., Duan T. (2012). Cross-immunoreactivity between bacterial aquaporin-Z and human aquaporin-4: Potential relevance to neuromyelitis optica. *The Journal of Immunology*.

[54] Vojdani A., Mukherjee P. S., Berookhim J., Kharrazian D. (2015). Detection of Antibodies against Human and Plant Aquaporins in Patients with Multiple Sclerosis. *Autoimmune Diseases*.

[55] Capsoni S., Ugolini G., Comparini A., Ruberti F., Berardi N., Cattaneo A. (2000). Alzheimer-like neurodegeneration in aged antinerve growth factor transgenic mice. *Proceedings of the National Acadamy of Sciences of the United States of America*.

[56] Rosenmann H., Grigoriadis N., Karussis D. (2006). Tauopathy-like abnormalities and neurologic deficits in mice immunized with neuronal tau protein. *JAMA Neurology*.

[57] Gebhard R., Huff C., Osborne M., Riegle L., Kelly-Worden M. (2015). Streptococcal Antibody Probe Crosses the Blood Brain Barrier and Interacts within the Basal Ganglia. *Open Journal of Pathology*.

[58] Bredesen D. E. (2014). Reversal of cognitive decline: A novel therapeutic program. *AGING*.

[59] Gill I., Kaur S., Kaur N., Dhiman M., Mantha A. K. (2017). Phytochemical Ginkgolide B Attenuates Amyloid-*β*1-42 Induced Oxidative Damage and Altered Cellular Responses in Human Neuroblastoma SH-SY5Y Cells. *Journal of Alzheimer's Disease*.

[60] Cai Z., Wang C., Yang W. (2016). Role of berberine in Alzheimer’s disease. *Neuropsychiatric Disease and Treatment*.

[61] Lakey-Beitia J., González Y., Doens D. (2017). Assessment of Novel Curcumin Derivatives as Potent Inhibitors of Inflammation and Amyloid-*β* Aggregation in Alzheimer's Disease. *Journal of Alzheimer's Disease*.

[62] Sathya M., Moorthi P., Premkumar P., Kandasamy M., Jayachandran K. S., Anusuyadevi M. (2017). Resveratrol Intervenes Cholesterol- and Isoprenoid-Mediated Amyloidogenic Processing of A*β*PP in Familial Alzheimer’s Disease. *Journal of Alzheimer's Disease*.

[63] Cacabelos R., Torrellas C., Carrera I. (2016). Novel Therapeutic Strategies for Dementia. *CNS & Neurological Disorders - Drug Targets*.

[64] Kumar A., Singh A., Ekavali (2015). A review on Alzheimer's disease pathophysiology and its management: an update. *Pharmacological Reports*.

[65] Ehret M. J., Chamberlin K. W. (2015). Current Practices in the Treatment of Alzheimer Disease: Where is the Evidence After the Phase III Trials?. *Clinical Therapeutics*.

[66] Zhao Y., Cong L., Jaber V., Lukiw W. J. (2017). Microbiome-Derived Lipopolysaccharide Enriched in the Perinuclear Region of Alzheimer’s Disease Brain. *Frontiers in Immunology*.

[67] Smith J. L., Bayles D. O. (2006). The contribution of cytolethal distending toxin to bacterial pathogenesis. *Critical Reviews in Microbiology*.

[68] Morales W., Weitsman S., Kim G., Marsh E., Chang C., Pimentel M. (2013). Tu2110 Circulating Antibodies to Cytolethal Distending Toxin B Correlate With the Development of Small Intestinal Bacterial Overgrowth in a Rat Model of Post-Infectious IBS. *Gastroenterology*.

[69] Pimentel M., Morales W., Pokkunuri V. (2015). Autoimmunity Links Vinculin to the Pathophysiology of Chronic Functional Bowel Changes Following Campylobacter jejuni Infection in a Rat Model. *Digestive Diseases and Sciences*.

[70] Rahmani A., Shoae-Hassani A., Keyhanvar P., Kheradmand D., Darbandi-Azar A. (2013). Dehydroepiandrosterone Stimulates Nerve Growth Factor and Brain Derived Neurotrophic Factor in Cortical Neurons. *Advances in Pharmacological Sciences*.

[71] Alderson R. F., Alterman A. L., Barde Y.-A., Lindsay R. M. (1990). Brain-derived neurotrophic factor increases survival and differentiated functions of rat septal cholinergic neurons in culture. *Neuron*.

[72] Yuan J., Huang G., Xiao Z., Lin L., Han T. (2013). Overexpression of *β*-NGF promotes differentiation of bone marrow mesenchymal stem cells into neurons through regulation of AKT and MAPK pathway. *Molecular and Cellular Biochemistry*.

[73] Halepoto D. M., Bashir S., Al-Ayadhi L. (2014). Possible role of brain-derived neurotrophic factor (BDNF) in autism spectrum disorder: Current status. *Journal of the College of Physicians and Surgeons Pakistan*.

